# Microbial Alterations and Risk Factors of Breast Cancer: Connections and Mechanistic Insights

**DOI:** 10.3390/cells9051091

**Published:** 2020-04-28

**Authors:** Sheetal Parida, Dipali Sharma

**Affiliations:** Department of Oncology, Johns Hopkins University School of Medicine and the Sidney Kimmel Comprehensive Cancer Center at Johns Hopkins, Baltimore, MD 21218, USA; sparida1@jhu.edu

**Keywords:** microbiota, microbiome, breast cancer, obesity, aging, estrogen, periodontal disease, xenobiotics, microbial metabolites

## Abstract

Breast cancer-related mortality remains high worldwide, despite tremendous advances in diagnostics and therapeutics; hence, the quest for better strategies for disease management, as well as the identification of modifiable risk factors, continues. With recent leaps in genomic technologies, microbiota have emerged as major players in most cancers, including breast cancer. Interestingly, microbial alterations have been observed with some of the established risk factors of breast cancer, such as obesity, aging and periodontal disease. Higher levels of estrogen, a risk factor for breast cancer that cross-talks with other risk factors such as alcohol intake, obesity, parity, breastfeeding, early menarche and late menopause, are also modulated by microbial dysbiosis. In this review, we discuss the association between known breast cancer risk factors and altered microbiota. An important question related to microbial dysbiosis and cancer is the underlying mechanisms by which alterations in microbiota can support cancer progression. To this end, we review the involvement of microbial metabolites as effector molecules, the modulation of the metabolism of xenobiotics, the induction of systemic immune modulation, and altered responses to therapy owing to microbial dysbiosis. Given the association of breast cancer risk factors with microbial dysbiosis and the multitude of mechanisms altered by dysbiotic microbiota, an impaired microbiome is, in itself, an important risk factor.

## 1. Introduction

The efforts of human microbiome projects and advances in culture-independent omics technologies have revolutionized our understanding of the relationship between humans and the commensals residing within our bodies. The human microbiome project was initiated in 2008, with the objective of understanding human beings as super-organisms, comprising human and over 100 trillion non-human microbial cells, whose collective genes exceed human genes by 150 times [1]. Trillions of microbes have co-evolved with humans, occupying different niches within the human body by supporting and augmenting metabolic processes for the sustenance of life; the normal gut microbiota alone account for 3.3 million genes [2]. It is imperative to investigate the interaction between the microbiome and the human genome, as microbiota can substantially manipulate the normal biological functions of the human body and have been found to influence the likelihood of major non-infectious diseases including diabetes, autoimmune diseases, inflammatory bowel disease (IBD) and various organ specific cancers. The human microbiome project primarily aimed to characterize the microbiota of five body sites including those of the skin, mouth, nose, colon and vagina. Intriguingly, a decade of research has also revealed the presence of unique and distinct microbial communities, even in organs previously considered “sterile or devoid of microbes” including the lungs [3,4,5], pancreas [6,7,8], prostate [9,10,11] and breasts [1,12]. An imbalance in the composition of these microbial communities is capable of supporting a carcinogenic transformation in these sites by altering immunological functions [13,14,15,16,17,18,19,20], energy harvest efficiency [21,22], the synthesis of small metabolites, which could function as signaling molecules [23,24,25,26], and the regulation of circulating levels of steroid hormones in the body [27]. Some of the known microbial species are capable of influencing DNA damage, genomic instability, mutations and epigenetic modifications [28,29]. It is interesting to note that the microbiota can affect Hanahan and Weinberg’s widely accepted hallmarks of cancer [30]. Moreover, several risk factors associated with breast cancer have a two-way relationship with microbiota, as they are modulated by microbial dysbiosis and can modulate microbiota. In this article, we review the relationship between several important breast cancer risk factors and microbial dysbiosis and also discuss the ways in which the human microbiota may influence breast cancer progression and responses to treatment.

**Search strategy and selection criteria:** A PubMed search was performed using various keywords, including cancer, breast cancer, microbiota, microbiome, breast cancer risk factors, cancer immunoregulation and microbial metabolites, in order to acquire original papers, population-based studies and review articles in English. All papers relevant to the importance of microbiota in breast cancer growth, progression, aggressiveness, metastasis and disease outcome were included. Papers and trials not directly related to the role of microbiota in breast cancer in general were excluded. All papers referred to are cited.

## 2. Microbiota in Breast Cancer

The connection between microbes and breast cancer was initially put forth by epidemiological studies querying the impact of antibiotic usage on breast cancer. Antibiotics can modulate the intestinal flora, resulting in the reduced metabolism of phytochemicals that may, in turn, lower their cancer-protective effects. On the other hand, a decrease in estrogen metabolism via the intestinal flora can lower circulating estrogen, lowering the risk of developing of estrogen receptor (ER)-positive breast cancer [31,32]. A case control study queried the use of antibiotics in 2266 women with breast cancer and 7953 healthy controls and inferred that antibiotic use was associated with a higher breast cancer incidence and the aggressiveness of the disease. However, a causal relationship with antibiotics and breast cancer was not determined [33]. A cohort of 2.1 million women with a follow-up of nine years showed a weak causal relationship between antibiotic use and breast cancer [34]. A weak association was also found between antibiotic use and higher tumor grade [35]. In a large study, including 3099 women with breast cancer and 12,396 matched controls, a non-causal dose-dependent relationship between antibiotic usage and increased breast cancer risk was noted [36]. A meta-analysis of five studies comprising of 13,069 breast cancer cases and 73,920 controls showed a slight increase in breast cancer risk in women who had ever used antibiotics vs. the never-used antibiotics group [37]. Frequent antibiotic use (≥4 antibiotics in one year) showed a modest elevated risk for second breast cancer events in a large study involving 4216 women [38]. The modulation of microbiota with antibiotic use has recently been associated with therapeutic outcomes in cancer patients undergoing immunotherapy regimens (reviewed in [39]). Collectively, these studies indicate that antibiotic usage may modulate breast cancer risk, although the establishment of a causal relationship requires additional studies, and the mechanistic links may be manifold, ranging from microbiota alterations to underlying disease conditions. Nonetheless, these epidemiological studies have sparked investigations to decipher the links between microbiota and breast cancer in a more directed manner.

Multiple recent studies have investigated the breast microbiota in cohorts comprising healthy volunteers, women with benign or malignant breast cancer and breast cancer survivors [25,26,40,41,42,43,44]. A study including 81 women from Canada and Ireland collected breast tissue from various locations within the breast, including areas close to the nipple as well as the chest wall and found the presence of bacteria in all locations. Diverse bacterial communities were present in breasts with Shannon’s diversity indices of 0.8–5.2, with an average of 3.6, which was quite comparable to oral and gut samples with diversity indices between 3.9 and 6.5. Organisms associated with pathogenesis in other organs, including *Streptococcus agalactiae*, *Enterobacteriaceae* and *Pseudomonas*, were also detected in breast tissue [40]. An examination of breast tumors and adjacent normal tissue from the same patient showed unique microbial communities associated with tumors as well as normal samples. Breast tumors had lower bacterial DNA loads in comparison to normal breast tissue and this correlated inversely with increasing tumor stages. Interestingly, unique microbiota alterations were noted in tumor and normal tissues, with the enrichment of *Sphingomonas yanoikuyae* in normal tissue and *Methylobacterium radiotolerans* in tumor tissue [45]. Both studies noted an abundance of members of Proteobacteria phylum in breast tissue which was distinct from other body sites where this phylum represents a small portion of the total bacterial load [40,45]. Analyses of paired breast tissue and breast skin samples showed the presence of distinct microbiota in breast tissue, especially the rare bacterial lineages [42]. An interesting question with respect to breast cancer microbiota is whether distinct microbiota alterations are associated with benign or malignant breast cancer. A comparison between adjacent normal breast tissue acquired from women with benign breast disease or with invasive breast cancer revealed significant differences in their microbiota. Breast tissue from women with invasive breast cancer revealed an abundance of some lowly abundant genera, including *Atopobium*, *Fusobacterium*, *Lactobacillus*, *Hydrogenophaga* and *Gluconacetobacter* [42]. The enrichment of specific genus-level taxa in adjacent normal breast tissue associated with malignant disease puts forth the notion that distinct microbiota alterations may be important for disease progression. Studies by Urbaniak et al. and Hieken et al. found a similar order of abundance of phyla-Bacteroidetes, Actinobacteria, Firmicutes and Proteobacteria in ascending order [40,42]. Since the breast ductal tree has 6–8 openings at the nipple area, which may allow microbes from the environment to inhabit the ductal system of the breast, it is plausible that nipple aspirate fluid contains microbes and that this may correlate with breast cancer. Indeed, the examination of nipple aspirate fluid (NAF) from women with a history of breast cancer or healthy volunteers showed that NAF from the breast cancer group was enriched with the genus *Alistipes*, whereas NAF from healthy volunteers contained an abundance of an unclassified genus from the *Sphingomonadaceae* family [41]. These studies showed the presence of distinct microbiota in breast tissue and highlighted the differences between study groups. Our group uniformly re-analyzed the raw data from the abovementioned studies in an attempt to find common candidate microbes across the data sets that could account for breast carcinoma [46]. Differences in community composition across data sets can be attributed to ethnicities, dietary habits, geography, lactation status, the method of sample collection and platform of sequencing and data analysis. The majority of breast microbiota are composed of Firmicutes, Bacteroides and Proteobacteria. Some interesting patterns were observed in microbiota composition, e.g., in one data set, 2.2% of the total bacterial count in the healthy breast tissue was *Lactobacillus*, whereas in cancerous tissue, it formed only 1.4% of the total bacterial count. It is noteworthy that *Lactobacilli* have been shown to be protective against breast cancer. Some microbes with known cancer-promoting functions were found to be enriched in breast tumors, e.g., breast cancer tissue overrepresented *Bacillus cereus*, a microbe known to metabolize progesterone and testosterone to 5-alpha-3,20-dione(5αP) and to induce the proliferation of breast cancer cells in vitro. Two important microbes, *E coli* and *Staphylococcus epidermidis*, known to induce double-stranded DNA breaks in HeLa cells, were found to be enriched in cancerous breast tissue. Breast cancer tissue samples were also found to harbor higher levels of *Fusobacterium* sp. Notably, *Fusobacterium* is a well-known cancer-promoting pathogen in colorectal cancer CRC [46]. Collectively, these studies not only demonstrate the presence of breast microbiota, but also show the association of distinct microbiota with breast cancer.

## 3. Risk Factors Associated with Breast Cancer

Broadly classified as modifiable and non-modifiable, several risk factors have been associated with breast cancer. Non-modifiable risk factors include gender, age, race, genetic susceptibility, exposure to radiation, family or personal history of breast cancer, high breast density, benign breast disease, early menarche, late menopause and steroid hormone levels, while obesity, a lack of physical activity, alcohol, oral contraceptive use/hormone replacement therapy, parity, breastfeeding, and periodontal disease are considered modifiable risk factors. Women are at a higher risk of developing breast cancer (one in eight women will develop breast cancer in their lifetime) in comparison to men (one in 883 men will develop breast cancer in their lifetime) [47,48]. Breast cancer risk increases with age and older women show a higher incidence of breast cancer. While the total number of breast cancer incidences is higher in white women, black women are at a higher risk of developing triple-negative breast cancer, and a more aggressive disease [49]. Several tools have been developed to assess the risk of developing breast cancer in women. Gail 1 included multiple risk factors, such as age at menarche, age, age at live birth of first child, family history of breast cancer, number of breast biopsies, number of biopsies showing atypical hyperplasia, and race/ethnicity. Later, it was modified to include the history of affected first degree family members (Gail 2), occupational physical activity level, education, body mass index (BMI), alcohol consumption status, leisure activities (h/wk) (absolute risk prediction model) and to include black women (the Women’s Contraceptive and Reproductive Experiences or CARE), and US Hispanic women for better risk prediction [50,51,52,53,54]. Breast and Ovarian Analysis of Disease Incidence and Carrier Estimation Algorithm (BRCA) probability tools (Ontario Family History Assessment Tool, BRCAPRO, Myriad I and II, and Manchester scoring system) were also developed to account for genetic risk factors [50,54,55,56]. Multiple risk factors have been defined for breast cancer incidence, but it is important to note that a large number of (~70%) women who develop breast cancer in their lifetime do not carry established risk factors. There is an immediate need to look beyond the usual suspects and incorporate newer aspects, such as microbial alterations. Recent studies have shown an association of some of the breast cancer risk factors, namely obesity, aging, estrogen exposure (modulated by early menarche and late menopause) and periodontal disease, with microbiota. Hence, in this section, we will review the microbial alterations related to obesity, aging, high estrogen levels and periodontal disease.

### 3.1. Obesity, Breast Cancer and Microbiota

Obesity is an important risk factor for breast cancer, not only because of its physiological effects on an individual, but also due to its steadily increasing worldwide prevalence. Approximately, 13% of the world population is obese and 36% adults in USA are obese [57]. Multiple studies and meta-analyses have reported a positive correlation between breast cancer and obesity [57,58,59,60,61,62,63,64,65,66,67]. Though the magnitude of its impact varies among studies, a strong positive correlation has been observed between high body mass index (BMI) and hormone receptor-positive breast cancer risk in postmenopausal women [57], while studies focusing on premenopausal women have shown a positive correlation, as well as a negative correlation, between high BMI and overall breast cancer risk [57,58,68,69,70]. In most studies, the risk of triple-negative breast cancer (TNBC) and inflammatory breast cancer (IBC) is positively associated with a higher BMI. In a case-control study with 68 IBC patients, IBC risk was 4 fold higher with BMI >26.65 [71]. IBC risk also increases between three and fivefold with obesity in postmenopausal women [71]. Overall, a poor prognosis and higher mortality have been observed in obese breast cancer patients, irrespective of menopausal status, breast cancer subtype and ethnicity (reviewed in [57]).

With the growing appreciation of the role of gut microbiota in metabolic processes, efforts are underway to decipher the interplay between the gut ecosystem, diet and obesity [24,72,73,74,75,76,77,78,79,80,81,82,83,84,85,86,87]. The idea was first put forth by immunologist Elie Metchnikoff in his disquisition ‘‘Essais optimistes’’, where he proposed that the products of microbial fermentation in the gut could cause systemic inflammation and obesity [80]. Though most of the studies on human microbiota have focused on its immune-mediated roles, a number of physiological functions independent of immune responses have also emerged. For example, many bacterial metabolites capable of inducing cellular signaling pathways have gained attention [15,23,73,88,89,90,91,92,93,94,95,96,97,98,99,100,101,102,103,104,105,106]. The descriptive profiling of gut microbiota has revealed a correlation between obesity and microbial classes in both humans and rodents [77,79,80,85,87,103,107,108,109]. In the obese state, an increase in the abundance of Firmicutes and a decrease in Bacteroidetes has been observed in most studies [77,79,87,108,110], except a few that report opposite results [21,111,112]. Within phylum Firmicutes, Mollicutes are most abundant in the obese state [87]. Most Firmicutes encode enzymes involved in lipid and carbohydrate metabolism. While some studies noted an increase in the levels of Actinobacteria and a decrease in Bacteroidetes, with no change in Firmicutes, others have seen an increase in *Lactobacilli* (phylum Firmicutes) in the obese state [77,111]. Again, among *Lactobacillus* species, a higher occurrence of *Lactobacillus reuteri* and lower levels of *Lactobacillus casei/paracasei* and *Lactobacillus plantarum* have been associated with obesity [113]. Interestingly, a similar increase in Firmicutes to Bacteroides ratio has been observed in breast cancer compared to normal breasts [45,46] and malignant breast cancer compared to benign breast disease as well [42,46]. In obese women, reduced Bacteroides and increased *Staphylococcus*, *Enterobacteriaceae* and *E. coli* has been observed with high BMIs [77]. The overrepresentation of *Staphylococcus*, *Enterobacteriaceae* and *E. coli* has also been observed in breast cancer tissue [46]. Overall, many microbial families and species enriched in the obese state are similar to those observed in breast cancer samples compared to normal breasts, suggesting a microbial link between obesity and breast cancer risk [46]. Though a direct association is yet to be found, obesity has also been shown to be influenced by certain groups of microbial commensals, which could be associated with long-term dietary choices [24,75,111,112,114].

Interestingly, it has been demonstrated that adiposity in humans as well as mice could be altered by fecal transplants and/or inoculation with specific bacteria and, moreover, that humanized germ-free mice mirror the donor’s adiposity [85]. While some studies have shown the extensive remodeling of microbial ecology being causally associated with obese phenotypes, suggesting the role of specific microorganisms, others highlight the importance of species-independent genes or pathways with common molecular functions [80,85]. The strongest evidence supporting a microbial association with obesity comes from the fact that germ-free mice demonstrate lower body fat mass despite increased food consumption compared to conventionalized mice [80,85]. Higher adiposity has also been observed in conventional mice on autoclaved normal diets, as well as high fat diets, compared to germ-free mice [80,85]. Intriguingly, colonizing germ-free mice with a combination of *Bacteroides thetaiotaomicron* and *Methanobrevibacter smithii* results in an increase in weight gain and adiposity [77,80,113]. The microbiota’s contribution to adiposity is, indeed, multifold and various hypotheses, including increased energy harvest and enhanced metabolism, have been put forward to explain the microbiota–obesity connection.

**Figure 1:**
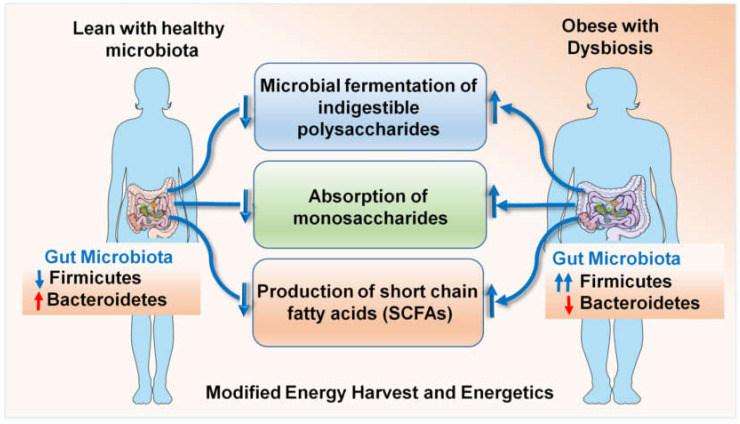
Microbial dysbiosis in obese states contributes to dysregulated energy harvest and energetics.

The enhanced energy harvest efficiency of an obese microbiota [77,87] occurs due to a culmination of multiple interdependent processes, including the microbial fermentation of otherwise indigestible polysaccharides, the absorption of monosaccharides and the production of short-chain fatty acids (SCFAs) such as butyrate, propionate and acetate [76,78,101,106,115] (Figure 1). SCFAs undergo hepatic conversion to form complex lipids and microbiome-mediated gene regulation promotes the storage of these complex lipids in the adipocytes. Mice deficient in G protein coupled receptor GPR41, a cell surface receptor for a variety of SCFAs [73], were found to be protected from obesity when lacking a normal gut microbiota but not in the presence of a conventional microbiota [24]. Microbiota-induced YY peptide expression also alters food intake [74]. Alternatively, this can be due to defects in normal leptin production driven by SCFAs in the adipose tissue [116], since GRP41-deficient mice do not produce normal levels of leptin [24]. Another proposed mechanism mediating obesity protection in germ-free mice is the lack of expression of fiaf/angptl4 in the intestinal lumen, which negatively regulates lipoprotein lipase, the enzyme responsible for maintaining lipid uptake into tissue [117]. Some classes, e.g., Firmicutes, have been described to be involved in fat metabolism and weight regulation [24,114] but to fully understand the association, it is necessary to conduct studies on germ-free mice and compare them with mono-colonized mice, followed by an evaluation of the underlying metabolic pathways. Experimental clarification about the extent to which this increased energy harvest can influence adiposity by overcoming the body’s homeostatic system has been provided by the elegant studies of Gordon and colleagues [118]. They have demonstrated that gut microbiota modification by fecal transplant can result in a 60% increase in total body adipose mass within 10 to 14 days in 8- to 10-week-old mice [118]; this corresponds to 4.7 to 7 years in a 20-year-old human being. Such fat gain is sufficient to transform a lean individual into a morbidly obese individual in short time, completely dysregulating the hormonal balance of the body. Gordon and colleagues colonized germ-free mice with *Bacteroides thetaiotaomicron*, which led them to the understanding that the gut microbiome modulates the gene expression required for vasculogenesis and angiogenesis, in addition to nutrient absorption, immune regulation and xenobiotic metabolism [119,120]. A multitude of interesting observations have put forth the relevance of microbial dysbiosis in the obesity–breast cancer axis. As such, obesity is one of the most important risk factors in breast cancer. The identification of specific taxa involved in the modulation of bodily fats can help develop a strategy to therapeutically prevent and treat obesity, thus reducing breast cancer risk. Clinical studies are investigating the microbiome connection to breast cancer aggravated by obesity [121]. One candidate organism that has gained importance in the recent past is *Akkermansia muciniphila*, which in under-represented in the gut of obese individuals. The loss of *A. muciniphila* causes the thinning of the mucus lining of the intestines, thus allowing lipopolysaccharide LPS and other endotoxins to leak into the circulation, causing chronic inflammation. A clinical trial examining *A. muciniphila* in obese breast cancer patients aged 60 to 70 years observed an association between BMI and *A. muciniphila* levels in early stages of breast cancer. Alpha diversity positively correlated with *A. muciniphila* levels [122,123]. Species richness, as well as fat mass, positively correlated with critical cytokine IL6 [123], suggesting that the gut microbial population is altered in the obese state, which can lead to chronic inflammation, which can worsen breast cancer outcomes.

### 3.2. Aging, Breast Cancer and Microbiota

The risk of being diagnosed with breast cancer increases with age, which is one of the non-modifiable factors. Breast cancer diagnosed in women over age 50 accounts for ~80% of all breast cancers and an exponential rise in invasive breast cancer and ductal carcinoma in situ has been observed up until menopause (age 50). The 10-year probability of invasive breast cancer incidence is 1.5% at age 40, increasing to 3% and 4% by age 50 and 70 respectively, leading to an overall risk of 13.2% or one in eight women. All breast cancer subtypes exhibit similar increases in age-specific incidence rates in premenopausal settings, while only ER-positive/PR-positive and ER-positive/PR-negative subtypes maintain increased age-specific incidence rates in postmenopausal settings [124]. Cellular and molecular changes associated with aging may render normal breast epithelial cells susceptible to malignant transformation [125,126]. Microbiome changes present another key aspect of the cancer–aging relationship, as the human microbiota is dynamic and it continuously evolves with age and exposure to environmental factors. An infant gut is mainly colonized with *Bifidobacterium infantis* and some related species, the only organisms capable of digesting the complex indigestible oligosaccharides of mother’s milk, which are indispensable for the baby’s energy requirements [127,128]. These bacteria also prevent the colonization of pathogens, since infants do not have a well-developed immune system, and set in motion the first immune response in a human body. As humans age, the microbiota co-evolves depending on dietary habits, drug usage and environmental factors. Firmicutes and Bacteroidetes are the major taxa present in the human gut and their relative ratio has been implicated in health and disease. Furet and colleagues analyzed the F/B ratio in infants, the adult and elderly population, and found that the F/B ratio was significantly higher in adults, but showed a drop in the elderly, showing no significant difference from infants [129]. Interestingly, the levels of genus *Bifidobacterium* was similar in adults and the elderly [129], suggesting that, although there is a depletion of microbial diversity in the elderly, which can slow down and prevent metabolism and the degradation of xenobiotics in a similar manner to infants, the elderly do not gain a protective immune environment as infants conferred by probiotics like *Bifidobacterium* do. *Bifidobacterium* is known to inhibit breast cancer progression [130] and has been proposed as a therapeutic strategy [130,131,132]. Although the microbial control of aging has been widely appreciated lately [133,134,135], it actually started with Elie Metchnikoff in the early 1900s. Aging has been found to be associated with a decline in the saccharolytic bacterial population and increased proteolytic bacteria in the gut [136]. Consequently, SCFAs in circulation are greatly reduced, impairing immune modulation. Aging sets in motion a process of chronic inflammation, increasing the levels of IL6, IL8 and TNFα [133,135,136]. Such a pro-inflammatory environment is conducive to the development of many cancers, including breast cancer. In a controlled study with 178 older adults, a diet rich in fiber resulted in higher abundance of Firmicutes in the gut, with a decline of inflammatory markers IL6, IL8, TNFα and CRP [135]. In addition, a fiber-rich diet contributes to the estrogen milieu by means of phytoestrogens [137], which bind to ERβ in the skeletal and central nervous system, accomplishing the functions of estrogens ceased due to lower estrogen levels in old age. In the breasts, phytoestrogens competitively bind to estrogen receptor alpha (ERα), preventing local estrogen signaling within an adipose-rich tissue environment. Based on the current knowledge, a supplementation with probiotic species like *Lactococcus lactis*, *Lactobacillus rhamnosus*, *Bifidobacterium adolescentis*, *Bifidobacterium longum*, *Lactobacillus plantarum*, *Bifidobacterium breve*, *Bifidobacterium lactis*, *Lactobacillus acidophilus*, *Lactobacillus casei*, and *Lactobacillus johnsonii* combined with a fiber-rich diet could be an effective strategy to prevent age-related diseases including breast cancer.

### 3.3. Higher Levels of Estrogens, Breast Cancer and Microbiota

**Figure 2:**
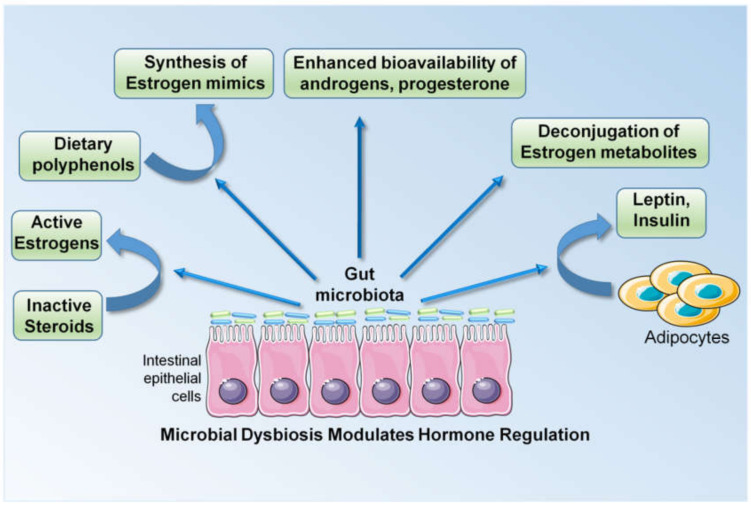
Changes in gut microbiota alter the levels of circulating hormones and cytokines/adipocytokines, which act as effector molecules, mediating the biological effect of dysbiosis.

Breast cancer growth and progression is largely regulated by steroid hormones and approximately 70% of breast cancers in postmenopausal women are estrogen receptor alpha (ERα)-positive, hence their sensitive to estrogen. Many triple-negative breast cancers, characterized by the absence of ERα, progesterone receptor (PR) and human epidermal growth factor receptor 2 (Her2), also possess ERβ and an androgen receptor (AR) [49]. An increased concentration of circulating estrogen and a longer duration of estrogen exposure are linked with a higher risk of breast cancer in postmenopausal women [138]. Late menarche (1-year delay) and late menopause (1-year delay) are associated with a 5% reduction and a 3% increase in the risk of developing breast cancer, respectively, highlighting the importance of the duration of estrogen exposure [139]. Multiple other risk factors such as the patient’s age at the birth of their first child, breastfeeding, high alcohol intake, and obesity in the postmenopausal stage are also associated with the level of circulating estrogens. Alcohol intake is associated with a 7% increase in the risk of developing breast cancer with a moderate intake (one unit/day) and the risk increases with higher alcohol intakes. Mechanistically, alcohol intake is associated with higher estrogen levels [140,141]. Another modifiable risk factor, obesity, is also associated with higher estrogen levels, owing to the increased aromatization of androstenedione in the adipose tissue of postmenopausal women. Obesity is linked with a 30% increase in the risk of breast cancer development [142,143]. It is now known that the microbiota is a critical regulator of estrogen in the body. Enteric bacteria, by virtue of their enzymatic activity, are capable of deconjugating estrogen metabolites and reintroducing them into the circulation by increasing their systemic levels. Bacterial glucuronidases and glucosidases are encoded by a group of enteric bacterial genes, collectively referred to as the estrobolome [144,145]. Multiple studies have shown links between microbiota and estrogen levels (reviewed in [145]). Estrogen undergoes hepatic deconjugation to form estrogen metabolites, possessing variable potency and bioavailability. These estrogen metabolites can either undergo biliary excretion or resorption into the circulation following glucuronidation and sulfonation [146]. The majority of these enzymatic reactions are catalyzed by intestinal bacteria. In addition, enteric microbes are capable of synthesizing estrogen mimics from dietary polyphenols, which can activate both ERα and ERβ signaling [146]. They are also known to regulate the bioavailability of steroid hormones in the tissue microenvironment. Gut microbes can metabolize and regulate the bioavailability of androgens, progesterone and testosterone. The sulfatase activity of certain gut commensals can potentially convert inactive steroids in circulation to active estrogen. The modulation of circulatory estrogen levels in the body by enteric commensals has been demonstrated by multiple population-based studies [147,148,149,150]. In addition to steroid hormones, breast cancer is also affected by hormones derived from adipose tissue, like leptin and insulin, which are also regulated by gut microbiota. Gut microbiota impacts various aspects of hormone regulation, resulting in the alteration of circulating levels of steroid hormones and cytokines that are known to increase the risk and progression of breast cancer (Figure 2).

### 3.4. Periodontal Disease, Breast Cancer and Microbiota

Meta-analyses of studies examining the association between periodontal disease and breast cancer have shown that periodontal disease is, indeed, an avoidable risk factor for breast cancer and can be considered for potential prevention strategies [151]. Many epidemiologic studies conducted over the years have shown an increase in the risk of esophageal, lung, pancreatic and breast cancers with periodontal disease. In 2016, an observational study on 73,737 postmenopausal women with periodontal disease was conducted [152]. Within a mean follow-up period of 6.7 years, 2124 cases of invasive breast cancer were recorded [152]. When adjusted for age, education, race/ethnicity, BMI, age at menarche and menopause, parity, maternal age at first birth, hormone use, alcohol consumption, physical activity and nonsteroidal anti-inflammatory drugs NSAIDs, the hazard ratio was 1.14 (CI = 1.04–1.26) and when additionally adjusted for smoking status, the HR was 1.1 (CI = 1.0–1.23) [152]. Non-smokers with a history of smoking within 20 years prior to study exhibited an HR of 1.36 (95% CI, 1.05–1.77) and for current smokers, HR was 1.32 (95% CI, 0.83–2.11) [152]. This study implies that periodontal disease positively correlates with breast cancer risk in postmenopausal women, especially former smokers [152]. Another study examined a cohort of 1337 postmenopausal women [153]. The mean follow-up period was 12.2 ± 4.2 years, during which there were 203 confirmed cancer cases. After adjusting for age and smoking, they did not find any significant association between periodontal disease and cancer in general. Only an increase in lung cancer risk was observed with HR 1.81, 95% CI 1.30–2.54 [153]. In another cohort study comprising of 65,869 periodontal disease cases between 54–86 years of age, 7149 cancer cases were recorded during a mean follow-up period of 8.32 years [154]. In relation to periodontal disease, total cancer risk increased by HR 1.14 (95% CI, 1.08–1.2). Breast cancer risk increased by HR 1.13 (95% CI 1.03–1.23) [154]. Similar associations were also observed in other cancers, such as lung cancer (HR, 1.31; 95% CI, 1.14–1.51), esophageal cancer (HR, 3.28; 95% CI, 1.64–6.53), gallbladder cancer (HR, 1.73; 95% CI, 1.01–2.95), melanoma (HR, 1.23; 95% CI, 1.02–1.48) and stomach cancer (HR, 1.58; 95% CI, 0.94–2.67) [154]. This study suggested that an increase in cancer risk with periodontal disease is not limited to any particular anatomical site [154]. In yet another study, a group of patients with periodontal disease, median age 49.6, were followed for 12 years and the study observed a 77% increase in the risk of any kind of cancer with periodontal disease [155]. The age-standardized incidence rate for breast cancer in women was 2.40, 95% CI 0.88–5.33 [155]. A study conducted on adult Brazilian women investigated 67 breast cancer patients at different stages and 134 controls [156]. Women with periodontal disease had a two to threefold higher odds ratio for breast cancer (2.72 1.18–6.27 .02) [156]. An association between the severity of periodontal disease and cancer incidence was also observed [154], which is largely mediated by oral microbial dysbiosis. Many pathogens associated with periodontal disease (PD) have also been identified with cancer lesions and are capable of promoting a pro-carcinogenic microenvironment [155]. Periodontal disease involves a complex interaction between host defense, bacteria and viruses [152,155]. A complex group of oral bacteria is known to form a biofilm on the teeth, which is capable of triggering an immune inflammatory response in the adjacent tissue. A systemic immune inflammatory response is also observed in some chronic cases of periodontal disease. Within a biofilm, quorum sensing is the primary mode of communication and involves the production and release of chemical signals called autoinducers [157], such as acylated homoserine lactones in the case of Gram-negative and oligo-peptides in Gram-positive bacteria [157]. The autoinducers regulate physiologic processes like symbiosis, virulence, competence, conjugation, antibiotic production, motility, and sporulation [157]. These chemical messengers may also trigger various signaling cascades in the host and contribute to cancer incidence.

## 4. Mechanistic Insights into the Microbiota-Cancer Connection

### 4.1. Microbial Metabolites as Effector Molecules Influencing Breast Cancer

Microbial residents of our body synthesize a plethora of metabolites including small molecules, fatty acids, vitamins, polyamines, hormones and ATP. In addition to circulating metabolites from the enteric microbes, it is now known that a community of microbes resides within the breast tissue, capable of synthesizing a myriad of signaling molecules. The local tissue of breast cancer patients has a significantly reduced population of *Methylobacterium*, which is known to synthesize phytohormones, cytokinin and auxin which have potent anticancer activities. Moreover, pathogenic strains of *E. coli* reside in the breast tissue of breast cancer patients, producing DNA-damaging colibactin. These strains cause double-stranded DNA breaks in vitro in HeLa cells [158]. A comprehensive list of metabolites produced by the human [25] microbiota is presented in Table 1 [22,27,159,160,161,162,163,164,165,166,167,168,169,170,171,172,173,174,175].

#### 4.1.1. Short Chain Fatty Acids (SCFAs)

SCFAs such as butyrate, propionate and acetate are the most important and well-studied microbial metabolites. SCFAs, produced in the small intestine, are the microbial messengers of the immune system. Bacteroides, which are more efficient in metabolizing dietary polysaccharides such as cellulose, produce acetate and propionate, while their less efficient partners, the Firmicutes, produce butyrate. Major immunologic functions within the gut are carried out by butyrate. It is the predominant energy source for colonocytes and is indispensable for the induction of T-regulatory cells. Comprehensive studies by Furusawa et al. and Smith et al. have demonstrated the role of microbial butyrate in the differentiation/maturation and maintenance of the homeostasis of FOXP3 expression in Treg cells, but not in their survival and proliferation [176,177]. Multiple studies have described the role of SCFAs in various aspects of immune regulation [14,16,177,178,179,180]. While most of the propionate is degraded in the liver, acetate is the only SCFA distributed throughout the circulation. SCFAs can induce both intracellular and extracellular signaling events [180]. They are also known to induce various GPCRs (GPR41, GPR43) or orphan fatty acid receptors [180]. In vitro, GPR41 and GPR43 have been shown to impede the invasion potential of MCF7 and MDA-MB-231 cells by inhibiting Hippo-Yap and MAPK signaling, respectively [181]. Interestingly, the binding of SCFAs to GPR41 has been shown to drive leptin synthesis. In addition, they can activate an array of cell surface receptors, including the niacin receptor GPR109A [182] and olfactory receptor 51E2, which are expressed on a variety of epithelial and immune cells [179,180]. The butyrate receptor GPR109A is expressed in healthy breast epithelial cells but is lost in breast cancers, irrespective of the cancer subtype [182]. Using multiple murine models, it has been demonstrated that butyrate-induced GPR109A activation inhibits cell survival and anti-apoptotic gene expression, while its deletion increases breast cancer incidence, as well as lung metastasis [182]. SCFAs can enter the cell via sodium channels (SLC5A8), where butyrate and propionate can inhibit histone deacetylase 1(HDAC1), 3 and histone acetyltransferases (HATs), thereby influencing transcriptional regulation. Butyrate is also known to antagonize PPARγ [180]. Unlike butyrate, acetate has been reported to be an instigator of cancers including liver, brain, prostate and breast cancer [183]. In cancer cells, acetate can serve as a source of nutrition required for lipid biosynthesis and can acetylate histones, leading to epigenetic modifications [183]. It can also lead to the considerable post-translational modification of proteins, altering their functions [183].

#### 4.1.2. Amino Acid Metabolism (Tryptophan, Arginine, Lysine)

Gut microbes such as *Lactobacilli* metabolize dietary tryptophan to various indole derivatives like indole-3-aldehyde. These indoles act as ligands for the Aryl hydrocarbon receptor (AhR) receptor. The AhR pathway is a central pathway of immune regulation in the gastrointestinal tract GI tract [180]. In breast cancer, a number of studies have suggested the important role of tryptophan metabolism and AhR signaling [184,185,186,187]. Amplified tryptophan metabolism and the upregulation of AhR receptors induce apoptosis resistance in breast cancer [186,187]. It is also known that the pregnane X receptor (PXR) gets modulated by microbial-specific indoles and affects the mucosal integrity via Toll-like receptors (TLRs). This is an interesting example of communication between the intestinal microbes and the PXR-TLR4 axis [188]. The activation of orphan nuclear receptors, PXR and steroid and xenobiotic receptors (SXRs), by endobiotics and xenobiotics, decreases growth and induces apoptosis in breast cancer cells [189,190]. Diamines, cadaverines or putrescines similar to spermines and spermidines, are products of the bacterial decarboxylation of amino acids lysine and arginine. These foul-smelling compounds maintain the pH of the environment making it conducive to bacterial growth. Multiple bacterial commensals, including *Shigella flexneri*, *Shigella sonnei*, *Escherichia coli*, and *Streptococci* encode enzymes LdcC and CadA are responsible for cadaverine biosynthesis (reviewed in [191]). Cadaverine has been shown to inhibit breast cancer cell proliferation, migration, invasion and stemness and to induce mesenchymal to epithelial transition both in vitro and in vivo via TAAR receptors [192]. It reduces tumor infiltration to the surrounding tissue, as well as reducing the distant metastasis of breast tumors [192]. Cadaverine biosynthesis is markedly reduced in early stages of breast cancer, rendering the developing tumors characteristically more glycolytic [192]. It could be attributed to the decreased biodiversity in breast cancers compared to healthy states.

#### 4.1.3. Secondary Bile Acids

Another important class of cancer-associated metabolites are secondary bile acids (SBAs), which are synthesized by all major classes of intestinal bacteria, including Firmicutes and Bacteroidetes, and are recognized as risk factor for liver and colon cancers. Primary bile acids are converted into SBAs by intestinal microbes in a series of successive deconjugation and dihydroxylation reactions. One secondary bile acid, lithocholic acid (LCA), has been shown to inhibit breast cancer cell proliferation and aggressiveness via TGR5. It induces the oxidative phosphorylation (OXPHOS) and tricarboxylic acid (TCA) cycle and inhibits epithelial to mesenchymal transition (EMT) and vascular endothelial growth factor (VEGF) levels in breast cancer [193]. As such, lower levels of LCA were detected in early stage breast cancer patients compared to healthy controls [193]. It was recently demonstrated that LCA, via its receptors, TGR5 and CAR, upregulates KEAP1, while downregulating NRF2 expression, which, in turn, leads to iNOS induction. Consequent protein and lipid oxidation cause cytotoxicity in breast cancer cells. With advanced breast cancer stages, a decrease in the microbial diversity and, hence, a decreased LCA level, results in the modulation of oxidative stress in the breast cancer cells, leading to a poor prognosis [191,194]. On the other hand, deoxycholate, another secondary bile acid, has been shown to promote mammary tumor cell survival by downregulating pro-apoptotic proteins [195].

#### 4.1.4. Bacteriocins/Peptides and Antibiotics

Bacteriocins are bioactive compounds produced by commensals, which are capable of inhibiting the growth and multiplication of other strains—notably, pathogens. These are valuable molecules, synthesized mostly by anaerobic gut commensals. Metagenome mining from datasets of the human microbiome project revealed codes for about 4875 bacteriocins of different types that are differentially distributed in various body sites [81]. Recently, antibiotic Lugdunin has been identified, which is synthesized by nasal commensal *Staphylococcus lugdunensis* and which prevents *S. aureus* colonization and infection [174]. While these microbial bacteriocins/peptides/antibiotics might not directly modify breast cancer risk, their therapeutic significance is tremendous. With advances in the understanding of dysbiosis, multiple carcinogenic strains are being discovered and eliminating pathogens to prevent/treat cancers or to improve treatment using broad-spectrum antibiotics seems lucrative, but collateral damage caused by broad-spectrum antibiotics is also well appreciated. Bacteriocins and peptides, on the other hand, have a narrow spectrum of activity, hence why they only inhibit closely related species. Additionally, they show higher activity at lower concentrations and do not induce antibiotic tolerance or resistance towards pathogens due to their unique mode of action [175]. Colonization with these microbial species can potentially eliminate pathogens, improving cancer outcomes. Many studies have described the therapeutic significance of bacterial species in treating cancers, mostly via immune modulation. However, inhibiting the colonization of pathogens by synthesizing small bioactive compounds could be a possible mechanism as well.

### 4.2. Microbiota Modulates the Metabolism of Xenobiotics

The most important consequence of the coevolution of humans with an army of commensals is the convergence of their enzymatic machineries in order to metabolize complex dietary components, pharmaceutical and non-traditional xenobiotics like environmental pollutants. The classes of microbial enzymes associated with xenobiotic metabolism include hydrolases, lyases, oxidoreductases and transferases [196], which are encoded by all major taxa. The mechanisms of the enzymatic reactions and interactions with hosts in the human body, e.g., the removal of glucuronide from chemotherapy drug Irinotecan by microbial hydrolases, increases their absorption and half-lives [196]. Lyases are responsible for breaking down complex polysaccharides. Microbes like *Eubacterium coprostanoligenes* reduce cholesterol to coprostanol, which is not absorbable and is subsequently excreted [197], keeping cholesterol levels in check. However, as sometimes encountered with dietary pollutants, the gut microbes can synthesize undesirable products. For example, food additive and artificial sweetener cyclamate is hydrolytically cleaved to form cyclohexamine, a potent carcinogen. Similarly, seemingly safe sweeteners, e.g., stevioside and xylitol, can also be metabolized by gut commensals via as yet undiscovered mechanisms [196]. The altered metabolism of xenobiotics by dysbiotic microbiota can influence cancer growth and progression.

### 4.3. Microbiota Induces Systemic Immune Modulation and Inflammatory Response in Breast Cancer

The gut microbiota is undoubtedly the stimulator of systemic immunity [198], which directly impacts breast cancer risk and aggressiveness. The induction of mammary adenocarcinoma was observed in response to enteric infection with *Helicobacter hepaticus* in Rag 2-deficient C57BL/6 Apc Min/+ mice [17]. Enteric infection with *Helicobacter hepaticus* induced a TNFα-dependent innate immune response, favoring mammary tumorigenesis, while mice lacking the adaptive arm of the immune system did not develop any mammary tumors in response to the bacterium [17]. In addition, the *H. hepaticus*-induced breast adenocarcinomas exhibited an increased infiltration of F4/80+ macrophages and the adoptive transfer of the T25+ TReg cells inhibiting mammary tumors [17]. However, the inhibition was much more efficient when TReg cells were subjected to a prior *H. hepaticus* challenge [17], suggesting that low-grade infection early in life can induce a tolerance to tumorigenesis. Enteric infection with pathogens like *Helicobacter pylori* prolongs the immune activation and, hence, leads to the sustained elevation of cycloxygenase 2 (COX-2) and prostaglandin 2 (PGE2) production, increasing the risk of cancer in susceptible individuals [199]. Interestingly, gut commensals can also initiate a systemic response that overrides pro-carcinogenic signals, preventing the progression of low-grade lesions to high-grade cancers in mice models by downregulating the systemic pro-inflammatory index in the epithelial tissue remote to the gut [198]. The microbiota regulates both inflammatory neutrophils and lymphocytes [200]. In breast cancer patients, crosstalk between microbes, systemic IL-6, and neutrophils has been observed [19,201]. The depletion of neutrophils by anti-Ly-6G antibodies resulted in the complete inhibition of tumor development in a bacteria-triggered mouse model of breast cancer [202,203]. Leukocyte infiltration is known to both prevent and promote tumor progression in a context-dependent manner [18,204]. In postmenopausal women, it is a determinant of breast cancer incidence and mortality [205]. Elevated neutrophil counts are known to be predictors of a poor prognosis in breast cancer patients [206]. A threefold higher early recurrence was observed in breast cancer patients with neutrophil to lymphocyte ratios higher than 2.5 (*p* < 0.001) [205] and negatively correlated with 5-year survival in 316 patients (*p* < 0.0001) [206]. In lymph node-positive patients, a higher ratio of lymphocytes (CD8+ effector T cells) correlated with lower relapse and death rates (*p* < 0.0001). Local inflammation in the breast tissue in response to enteric infection suggests a retrograde translocation of microbes from the gut to the mammary tissue, which potentially increases systemic inflammation, thereby promoting breast tumorigenesis [202]. Commensal microbes also play an important role in modulating therapy response. CD8+ T cells, or natural killer (NK) cells, have been found to be the most potent in eliminating breast tumor cells [207]. In a healthy gut, contact with the commensals *Sphingomonas* of Proteobacteria phylum [207,208] induces effector CD8+T cell maturation. In a dysbiotic state, microbiota-mediated CD8+T cell maturation and CD8+ antitumor cytotoxicity is hampered [208]. The microbiota can actively hinder the therapy response in breast cancer patients via immune modulation, since an inflammatory tumor microenvironment and an erroneous immune response is known to induce chemotherapy resistance in breast cancer [204]. Overall, human microbiota induce systemic immune modulation and inflammation and a dysbiotic microbiota may modulate breast cancer initiation and progression by manipulating various components of the host’s immune system.

### 4.4. Microbial Dysbiosis May Modulate Response to Cancer Therapy

Evidently, a healthy microbiota is indispensable for an optimum therapeutic response and dysbiotic microbiota could be the underlying reason for variable response to similar therapeutic strategies in different patients. Approximately forty chemotherapeutic drugs have been shown to be metabolized by the gut microbiota [196]. Some of the most commonly used cancer drugs known to be regulated by the microbiota include cisplatin, oxaliplatin, doxorubicin, cyclophosphamide, and misonidazole [209]. Diverse phyla are known to mediate drug metabolism via reactions like proteolytic degradation, isoxazole scission, denitration, deconjugation, acetylation/deacetylation, amine formation and/or hydrolysis, as well as by physical adherence to the drugs [46]. The gastrointestinal microbial population is also known to influence the response to radiation and immunotherapy [209]. Gut commensals induce tumor-associated myeloid cells in order to synthesize inflammatory cytokines like TNFα and IL1β in a TLR4-dependent manner, thus influencing adoptive T cell activation and, in turn, determining the efficacy of immunotherapy. Studies have also shown that, compared to germfree animals, conventional mice are more prone to ionizing radiation-induced DNA damage in peripheral leukocytes [210]. Moreover, the response to radiotherapy varies at different times during the day in concert with gut microbial metabolic activity, suggesting a potential relationship between the two [210]. Chemo and radiotherapy-associated systemic and organ toxicities are responsible for significant morbidities and are thought to be regulated by the microbiota. The association between microbiome and therapy-associated toxicities was elegantly described by the Translocation, Immunomodulation, Metabolism, Enzymatic Degradation and Reduced Diversity (TIMER) hypothesis [211]. Most gastrointestinal toxicities are known to be regulated by bacterial GUS genes encoded by the majority of gut commensals, and efforts to develop inhibitors of bacterial GUS are underway, with the hope of preventing chemotherapy-associated GI toxicities [212]. Certain probiotic *lactobacillus* strains have proved to be effective in preventing toxicities in colorectal cancers [213].

### 4.5. Beneficial Bugs as Drugs

In recent years, multiple studies have utilized probiotic bacterial strains for the prevention and treatment of breast cancer growth and metastasis, as well as improving the response to chemotherapy and immunotherapy (reviewed in [214,215,216,217]). *Lactobacillus reuteri*, a bacterium found in breast milk, is well known for its immunomodulatory effects and has been shown to inhibit the proliferation of breast cancer cells [218]. In a series of studies using multiple mouse models, it has been demonstrated that a healthy microbiota can be helpful in overcoming genetic, as well as environmental (diet-induced), predisposition to breast cancer development [13]. Using a Western diet-induced outbred Swiss mouse model and an FVB strain Her2-mutant spontaneous breast cancer model, it has been shown that oral administration of *Lactobacillus reuteri* inhibits mammary tumorigenesis by CD4+CD25+ lymphocyte stimulation [13]. In a Western diet-induced model, *L. reuteri* administration resulted in the inhibition of mammary atypical hyperplasia, with the restoration of FOXP3-positive cells in mammary lymph nodes, which are depleted in the absence of probiotic treatment, as well as a decrease in mast cell population [13]. *L. reuteri* administration also resists diet-induced obesity, with higher systemic levels of IL10 and lower levels of IL17 in mice [13]. In a transgenic breast cancer model, a reduced tumor burden and increased disease-free survival is observed when orally treated with *L. reuteri* [13]. In tumor-bearing mice, an increase in intra-tumoral apoptosis, necrosis and the downregulation of NFκB and c-Jun pathways in the neoplastic cells is also evident [13]. The adoptive transfer of CD4+CD45RBloCD25+ cells from mice exposed to *L. reuteri* is sufficient to regress tumors in a transgenic breast cancer model [13]. Interestingly, a transgenerational study demonstrated an increased risk of breast cancer and obesity in the second-generation progeny of mice that were given a modern Western diet. Interestingly, these mice exhibited gut microbiota changes that were carried across generations [219]. However, when the second-generation progeny was administered with a continuous dosage of *L. reuteri*, the cancer risk significantly reduced [219]. Interestingly, the administration of milk fermented with *Lactobacillus casei* CRL431 in mice before tumor injection inhibited tumor growth, angiogenesis and lung metastasis by reducing the infiltration of macrophages into the tumor, as well as lungs, while enhancing the CD4+ and CD8+ antitumor immune response. Probiotic *Lactobacillus casei* CRL431 delays tumor growth, even when it is administered after tumor detection [220]. Live cells, heat-killed cells, as well as the cytoplasmic fractions of *Enterococcus faecalis* and *Staphylococcus hominis* microbes isolated from human milk, all exhibit anti-breast cancer potential. While all the fractions caused a significant reduction in the proliferation of breast cancer cells, *Enterococcus faecalis* exhibited a better dose and time response [221]. *Lactobacillus acidophilus* has been shown to improve the survival of breast tumor-bearing mice by inducing increased immune cell proliferation and interferon γ (IFNγ) production, while downregulating IL4 production [20]. Cultured supernatants from *Lactobacillus crispatus* and *Lactobacillus rhamnosus* induce cell death in the triple-negative breast cancer cell line MDA-MB-231 and downregulate hypoxia-associated genes HIF1α, HSP90 and SLC2A1 [222]. *L. rhamnosus GG* has also been shown to reduce GLUT 1 expression in MDA-MB-231 cells. GLUT 1 inhibition can prevent metastasis via matrix metallopeptidase 2 (MMP-2) and JNK pathways. The nano delivery of *Lactobacillus brevis* improved NK cell activity and IFN-γ and IL-17 levels, inhibiting metastasis to vital organs, in a mouse model of breast cancer [215]. Together, these preclinical studies have formed a basis for clinical studies. Few ongoing clinical trials are investigating the impact of beneficial bacteria in breast cancer patients (Table 2). Subjects with stage I–III breast adenocarcinoma will be given a probiotic (Prima Defense Ultra containing 13 species of beneficial bacteria) for 2–4 weeks and the mean number of cytotoxic T lymphocytes (CD8+ cells) will be evaluated (ClinicalTrials.gov identifier: NCT03358511). An interventional double-blind, randomized, placebo-controlled pilot study is currently investigating the impact of probiotic natural health product RepHresh Pro-B, containing *Lactobacillus rhamnosus GR-1* and *Lactobacillus reuteri RC-14*, on breast microbiota and inflammation markers (ClinicalTrials.gov identifier: NCT03290651). Another randomized study is investigating the impact of MRx0518 (a proprietary strain of bacterium) on breast tumor biomarkers and overall survival (ClinicalTrials.gov identifier: NCT03934827). While in vitro and in vivo studies have highlighted the potential benefits of probiotics in the prevention and treatment of breast cancer (reviewed in [216,217]), large-scale clinical studies are needed to prove the benefits of adding beneficial bioactive strategies to the existing repertoire of drugs as combination strategies.

## 5. Conclusions

**Figure 3:**
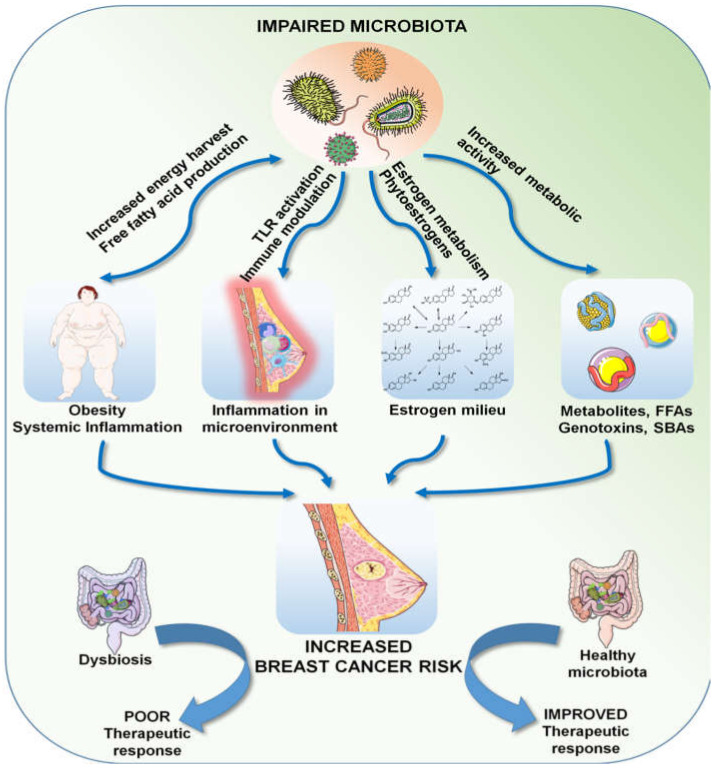
Multifaceted effects of a dysbiotic microbiota.

Breast cancer is a multifactorial disease, resulting from the interaction between genetic, epigenetic and environmental factors. Owing to the vastness of the metagenome, we are only beginning to understand its role in health and disease in general. Microbial dysbiosis in relation to the many established risk factors of breast cancer and its role in treatment response has been observed and described by many investigators. Genetics undoubtedly plays an important role in cancer incidence, but it only accounts for less than 10% of breast cancer cases, suggesting that environmental factors and the metagenome can play bigger roles in breast cancer incidence and progression. We are a long way from completely understanding the microbiota–breast cancer connection, but the current knowledge indicates a magnitude of roles; in a nutshell, an impaired microbiota can directly affect adiposity by altering energy harvest efficiency and synthesizing free fatty acids, which, in turn, can lead to hormonal imbalance. Optimum levels of steroid hormones, most importantly estrogen, are largely dependent on the metabolic activity of the gut microbiota, which are the central regulators of systemic immunity. Communities of organ-specific microbial commensals are also known to produce numerous active metabolites, including virulence factors, toxins, fatty acids, vitamins and cofactors, potent enough to activate various signaling cascades. All of these factors taken together can contribute to the overall risk of breast carcinogenesis. Moreover, the human microbiota can determine the response to cancer therapy by various mechanisms, as described above, and thus can positively or negatively affect the outcome (Figure 3). The microbiota/microbiome therefore deserves a special focus in our quest to understand breast cancer and the search for superior strategies to deal with it.

## Figures and Tables

**Table 1:** Microbes produce various metabolites that influence multiple biological functions.

Biological Process			Microbial Species	Reference
**Carbohydrate metabolism**				
**Parent carbohydrate**		**Metabolite**	Most gut organisms *Eubacterium hallii* *Roseburia* *Coprococcus catus* *Megasphera elsdenii* *Veillonella* spp *Ruminococcus obeum* *Bacteroidetes* *Eubacterium rectale* *Eubacterium hallii* *Roseburia* spp *Coprococcus* spp *Faecalibacterium prausnitzii* *Bifidobacterium* spp *Lactobacillus* spp *Methanobrevibacter smithii* *Desulfovibrio spp*	[159,161,163,168] [22]
Polysaccharides Oligosaccharides Resistant starch Mucins		Acetate Propionate Butyrate Lactate CH_4_ H_2_S		
**Fat metabolism**			*Eubacterium siraeum* *Roseburia faecis* *Roseburia intestinalis* *F. prausnitzii* *Bifidobacterium* *Propionibacterium* *Lactobacillus* spp *Bifidobacterium breve* **Propionibacterium freudenreichii** *Roseburia inulinivorans* *Roseburia hominis* *Butyrivibrio fibrisolvens* *Clostridia Peptostreptococci* *Peptococci*	[27]
**Linoleic acid** Conjugated lineloic acids				
Stearic acid				
**Fatty acids** Short chain fatty acids(SCFA) Branched chain fatty acid(BCFA) (Isobutyrate from valine; 2-methylbutyrate from isoleucine; Isovalerate from leucine)				
**Protein metabolism**			*Akkermansia* *Bifidobacterium*	[166,172]
**Bile metabolism**		Cholic acid Chenodeoxycholic acid Deoxycholic acid Lithocholic acid Ursodeoxycholic acid	*Bacteroides* *Bifidobacterium* *Clostridium* *Lactobacillus* *Listeria* *Clostridium* *Eubacterium* *Bacteroides* *Clostridium* *Egghertella* *Peptostreptococcus* *Ruminococcus* *Eubacterium*	[170]
**Vitamin synthesis**		Biotin Cobalamin Folate Nicotinic acid Panthothenic acid Pyridoxine Riboflavin Thiamine	*Bacteroidetes* (Most important) *Fusobacteria* *Proteobacteria* *Firmicutes* *Actinobacteria*	[165,166]
**Phytochemical metabolism**				
**Phytochemical** Flavonoids Lignans Ferulic acid Isofllavones Caretenoids Glucosinolates Isothiocyanates Stilbens Organosulfur compounds	**Process** Deglycosylation Ring fission Dehydroxylation		*Clostridium* spp. *Eubacterium ramulus* *Lactobacillus mucosae* *Enterococcus faecium* *Finegoldia magna* *Veillonella* sp *Slakia isoflavoniconvertens* *Slakia equolifaciens* *Adlercreutzia equolifaciens*	[166,171,173]
**Antibiotics**			*Staphylococcus lugdunensis* *Pantoea agglomerans* *P. Vagans* *Clostridium* sp.	[172,173]
Lugdunin Herbicolin I Andrimid Polykitide synthase				
**Bacteriocins**			*Lactococcus lactis* *Butyrivibrio fibrisolvens* *Pedicoccus acidilactici* *Lactobacillus sakei* **Lactobacillus acidophillus** *Enterococcus faecium* *Lactobacillus helveticus* *Enterococcus* sp. *Lactobacillus plantarum*	[85,174,175]
Nicin Lacticin 3147 Butyrivibriocin Pediocin Sakacin A Lactacin F Lactococcin G Enterolysin Helvecticin I Enterocin I F4-9 Glycocin F				

**Table 2:** Bugs as drugs.

Bug	Animal Model	Mode of Action	Reference
*Lactobacillus reuteri*	FVB strain Her2	Inhibits mammary tumorigenesis by CD4+CD25+ lymphocyte stimulation	[13]
	Swiss mice		
*Lactobacillus casei* CRL431	4T1 syngeneic breast cancer model in Balb/C mice	Inhibits tumor growth, vascularization and lung metastasis in mice by reducing infiltration of macrophages into the tumor and; enhances CD4+ and CD8+ antitumor immune response	[220]
*Lactobacillus casei*	4T1 syngeneic breast cancer model in Balb/C mice	Reduces breast cancer incidence	[216]
*Shirota*			
*Lactobacillus acidophilus*	4T1 syngeneic breast cancer model in Balb/C mice	Improves survival of breast tumor bearing mice	[20]
		Increased immune cell proliferation	
		Increased IFNγ production	
		Decreased IL4 production	
*Lactobacillus brevis*	4T1 syngeneic breast cancer model in Balb/C mice	Inhibits metastasis to vital organs	[215]
		Improves NK cell activity	
		Increased IFN-γ	
		Increased IL-17 levels	

## Abbreviations

IBD	Inflammatory bowel disease
BMI	Body mass index
TNBC	Triple-negative breast cancer
IBC	Inflammatory breast cancer
E coli	Escherichia coli
SCFA	Short chain fatty acids
IL	Interleukin
TNF	Tumor necrosis factor
CRP	C reactive protein
ERβ	Estrogen receptor beta
PD	Periodontal disease
NSAID	Non-steroidal anti-inflammatory drugs
HR	Hazard ratio
ERα	Estrogen receptor alpha
AR	Androgen receptor
PR	Progesterone receptor
Her2	Human epidermal growth factor receptor 2
GPCR	G-protein coupled receptor
HAT	Histone acetyltransferases
AhR	Aryl hydrocarbon receptor
GI	Gastrointestinal
SBA	Secondary bile acids
LCA	Lithocholic acid
TCA	Tricarboxylic acid
EMT	Epithelial to mesenchymal transition
VEGF	Vascular endothelial growth factor
COX-2	Cyclooxygenase 2
PGE2	Prostaglandin E2
TLR	Toll-like receptors
TIMER	Translocation, Immunomodulation, Metabolism, Enzymatic degradation and Reduced diversity
